# Immunopathology of SARS-CoV-2 Infection: Immune Cells and Mediators, Prognostic Factors, and Immune-Therapeutic Implications

**DOI:** 10.3390/ijms21134782

**Published:** 2020-07-06

**Authors:** Alessandro Allegra, Mario Di Gioacchino, Alessandro Tonacci, Caterina Musolino, Sebastiano Gangemi

**Affiliations:** 1 Division of Hematology, Department of Human Pathology in Adulthood and Childhood “Gaetano Barresi”, University of Messina, 98125 Messina, Italy; aallegra@unime.it (A.A.); cmusolino@unime.it (C.M.); 2 COVID Centre AOU Policlinic G. Martino, 98125 Messina, Italy; 3 Department of Medicine and Science of Ageing, G’ d’Annunzio University, 66100 Chieti, Italy; 4 Institute for Clinical Immunotherapy and Advanced Biological Treatments, 65100 Pescara, Italy; 5 Clinical Physiology Institute, National Research Council of Italy (IFC-CNR), 56124 Pisa, Italy; atonacci@ifc.cnr.it; 6 School and Operative Unit of Allergy and Clinical Immunology, Department of Clinical and Experimental Medicine, University of Messina, 98125 Messina, Italy; gangemis@unime.it

**Keywords:** SARS-CoV-2, immune response, cytokine storm, IL-6, prognostic factor, T lymphocyte

## Abstract

The present is a comprehensive review of the immunopathology of Covid-19. The immune reaction to SARS-CoV-2 infection is characterized by differentiation and proliferation of a variety of immune cells with immune mediator production and release, and activation of other pathogen resistance mechanisms. We fully address the humoral and cellular immune changes induced by the virus, with particular emphasis on the role of the “cytokine storm” in the evolution of the disease. Moreover, we also propose some immune alterations (i.e., inflammatory parameters, cytokines, leukocytes and lymphocyte subpopulations) as prognostic markers of the disease. Furthermore, we discuss how immune modifying drugs, such as tocilizumab, chloroquine, glucocorticoids and immunoglobulins, and blood purification therapy, can constitute a fundamental moment in the therapy of the infection. Finally, we made a critical analysis of a number of substances, not yet utilized, but potentially useful in SARS-CoV-2 patients, such as IFN lambda, TNF blockers, ulinastatin, siponimod, tacrolimus, mesenchymal stem cells, inhibitors of mononuclear macrophage recruitment, IL-1 family antagonists, JAK-2 or STAT-3 inhibitors.

## 1. Introduction

In December 2019, an epidemic provoked by Coronavirus disease 2019 (COVID-19) arose in Wuhan, Hubei Province, China. As of June 20, 8,700,000 COVID-19 cases were reported worldwide. More than 450,000 patients died from infection with this new virus called Severe Acute Respiratory Syndrome Coronavirus 2 (SARS-CoV-2).

SARS-CoV-2 belongs to the Coronaviridae family and is correlated to the subgenus Sarbecovirus. This is an enveloped virus comprising a single-stranded positive sense RNA viral genome. Virions are spherical, with the spiked glycoprotein inserted in the envelope [1]. In other viruses of the same family, this protein has been demonstrated to connect to host cellular receptors and to facilitate membrane fusion [2].

After entering the lungs by respiration, SARS-CoV-2- stimulates the activity of immune cells, increases cytokine production, and actives other pathogen resistance mechanisms.

Viral RNAs are identified by the innate immune system via three groups of pattern recognition receptors: RIG-I-like receptors (RLRs), NOD-like receptors (NLRs), and Toll-like receptors (TLRs), which stimulate the production of interferon (IFN) and trigger anti-viral effectors such as T CD8 + cells, Natural Killer (NK) cells, and macrophages [3,4].

Cytotoxic T lymphocytes (CTLs) are stimulated after identifying infected cells presenting the viral antigens as portions of surface antigen-MHC-I complexes. Efficacious presentation is determined by the correct harboring of antigens by MHC-I molecules through hydrogen bonds and salt–bridge relations that permit great affinity with higher specificity [5]. An immunoinformatic method was employed to recognize major CTL and B cell epitopes in the SARS-CoV-2 surface glycoprotein. The authors recognized five different CTL epitopes, three sequential B cell epitopes and five discontinuous B cell epitopes in the viral surface glycoprotein [6].

SARS-CoV and MERS-CoV infections are characterized by fast and robust initial virus replication with late IFN generation, resulting in disproportionate inflammatory host responses provoking grave lung alterations [7,8]. In the fight between the virus and the human body, the immunity of the subjects reduces, and the virus virulence augments [9]. This causes edema and congestion of the lung, thickening of the interstitial tissue, and augmented exudation in the alveolar space able to cause respiratory failure.

The purpose of this review is to analyze cellular and humoral immune changes induced by SARS-CoV-2 and to propose the possibility that such immune changes could be used as prognostic markers of the disease. Finally, we critically consider the various immuno-modifying drugs useful in the treatment of Covid-19 and underline how the immunotherapeutic approach is of fundamental importance for SARS-CoV-2 infection.

## 2. Immunopathology of SARS-CoV-2 Infection

### 2.1. Lymphocyte Subpopulations

Subsets of CD4+ T cells, CD8+ T cells, B cells, and NK cells play a central role in the functioning of the immune system. Several reports have studied the diverse lymphocyte populations in subjects with SARS-CoV-2 infection. 

Lymphocyte populations were studied in 44 subjects on admission. The total amount of T cells, B cells and NK cells was significantly reduced in infected group as T cells and NK cells were below the normal range, while B cells were within the lower quantity of the reference values. T cells are the most altered by the viral infection, approximately half the lower reference limit. However, the function of CD4+, CD8+ T cells, and NK cells was within normal limits in this study, as suggested by PMA/Ionomycin-triggered IFN-γ positive cells in these three populations. Moreover, examining the various subsets of T cells, the authors evidenced that both helper (CD3+CD4+) and cytotoxic T cells (CD3+CD8+) were below the normal range in subjects with COVID-19, with the T helper/suppressor ratio (Th/Ts) within normal limits. Furthermore, subjects with SARS-CoV-2 infection showed lower numbers of regulatory T cells (Treg) (CD3+CD4+CD25+CD127low+), and this reduction was especially evident in critical patients. A decrease in naïve (CD45RA+CD3+CD4+CD25+ CD127low+) and induced (CD45RO+CD3+CD4+CD25+CD127low+) regulatory T cells was also evident in critical patients, although the data did not reach statistical significance in comparison to other groups of patients [10]. 

These data have been substantially confirmed by other studies showing lymphocyte numbers below the normal limit in most subjects. CD+, CD8+, NK T cells and B lymphocytes were all reduced in infected subjects. The presence of comorbidities significantly modified the quantity of CD8+ T cells [11].

Indeed, all studies already agree in showing that T cell subset alterations strongly correlate with inflammatory condition. In particular, the quantity of CD8+ T cells was negatively correlated, while the CD4+/CD8+ ratio positively correlated with C-reactive protein (CRP), erythrocyte sedimentation rate (ESR), and IL-6.

Infected subjects who responded positively to the treatment showed an increase in CD8 + T cells and B cells, while no relevant changes in any lymphocyte subset were reported in unresponsive patients. Thus, lymphocytes and their subsets, mainly CD8+ T cells, might be a possible marker for clinical efficacy of treatments for SARS-CoV-2 infection [12].

Specific studies were performed for the analysis of CD4 + T lymphocytes. It is worth knowing that the differentiation of naïve CD4+ T-cells into effector and memory population is one of the most essential aspects of T-cell-mediated immunity [13], and the equilibrium between the naïve and memory CD4+ T cells is essential for maintaining an effective immune response. Such balance is altered in COVID-19 critical patients with expansion of naïve CD4+ T-cell subset and reduced memory cell percentage further showing the severity of the immune system impairment in these subjects. SARS-CoV-2 RNA load and lymphocyte amount and CD4+ T and CD8+ T lymphocyte quantity were observed to be negatively correlated [13]. These results suggest that the decrease in lymphocytes and their subsets, directly affected by the viral load, was closely correlated to disease evolution [14].

As for other lymphocyte subsets, a peripheral flow cytometry study demonstrated an increase in the number of Th17 cells, which differentiate from Th0 cells mainly stimulated by IL-6 and IL-23 [14]. 

Differently from other researchers, Cossarizza et al. demonstrated that the infection affects not only the number of lymphocytes but also their functions. They evidenced significant differences in the generation of cytokines between CD8 + T cells of patients with infectious pneumonia and healthy donors matched for age and gender. Indeed, most of the CD8+ T cells of these subjects were capable of generating Granzyme B but not INF-γ or TNF-α [15]. Moreover, Diao et al. demonstrated that the surviving T cells appear functionally exhausted [9].

Interesting data to explain the ultimate mechanism by which viral infection causes a reduction in various cell populations, apart from the obvious antigenic stimulation, come from transcriptomic studies. Xiong et al. demonstrated that, in bronchoalveolar lavage fluid, numerous significantly altered genes are related to apoptosis and P53 signaling pathways, comprising GTSE1, RRM2, CTSL, CTSB, DDIT4, CCNB1, RRAS, CDK1. CTSD, STEAP3, BIRC5, TNFSF10, CTSZ, NTRK1, IGFBP3, CCNB2, and TP53I3. Remarkably, TP53, an essential gene in the process of programmed cell death, exhibits an increasing trend, suggesting that peripheral blood mononuclear cells’ decrease may be due to an increase in programmed cell death [16].

### 2.2. Immune Mediators in Patients with SARS-CoV-2 Infection

Cytokines are known to have a crucial role in the immunopathology of viral infection. A ready and well-organized innate immune response represents the first protection against viruses. On the contrary, an altered and disproportionate immune response may provoke a severe immune-mediated injury [8,17,18].

Elevated concentrations of IL-1β, IFN-γ, IP-10, monocyte chemoattractant protein 1 (MCP-1) and IL-17 have been reported in SARS-CoV-2 subjects. These pro-inflammatory cytokines may stimulate the T-helper type 1 (Th1) cell response [19,20]. Moreover, serum concentrations of IL-2R and IL-6 in these subjects are positively correlated with the severity of the disease [21]. Furthermore, TNF-alpha, granulocyte colony-stimulating factor, MCP-1, IP-10, and macrophage inflammatory protein-1α were higher in patients in the intensive care unit (ICU) compared to infected subjects from general wards, confirming that cytokine generation is positively correlated with disease gravity [19].

Yang et al. studied clinically moderate and severe Covid-19 patients; they performed a multiplex screen for several cytokines and interrelated these data with viral loads and clinical features. They displayed a relevant increase of 14 cytokines in SARS-CoV-2-infected patients compared to healthy controls. Moreover, augmented concentrations of three of these proteins (IL-1 receptor antagonist, CCL7 and CXCL10) were positively correlated with viral load, the extent of lung damage and fatal prognosis [22]. 

Quantitative and qualitative differences in cytokine production characterizing different immune responses may justify different outcomes in specific categories of COVID-19 patients. Interestingly, it is now recognized that children and pregnant women generally have a mild disease after SARS-CoV-2 infection if not a fully asymptomatic one [23]. These patients are characterized by an immune response skewed toward a Th2 profile (ruled by the T-helper Type 2 cells), with specific generation of related cytokines like IL-4 and IL-10, while, the generation of Th1 pro-inflammatory cytokines typically characterizes SARS-CoV-2 infection. Therefore, it could be of interest to analyze whether profiling immune cells for their capability to generate Th1 or Th2 cytokines could be beneficial for the management of SARS-CoV-2-infected patients.

Several studies have carefully analyzed IL-6 plasma concentrations during COVID-19, also for possible immediate therapeutic implications. Actually, whether increased IL-6 concentrations are disadvantageous or favorable in infected patients is still unclear. Indeed, in experimental models, IL-6 can either reduce or increase viral proliferation [24]. However, most studies tend to consider increased IL-6 as a negative factor in patients with SARS-CoV-2 pneumonia. IL-6 can inhibit CD8+ cytotoxic T-cells by reducing their production of IFN-γ. Moreover, IL-6 can block the cell-mediated antiviral response during a cytokine storm by inhibiting specific cytokine signaling such as SOCS-3 and increasing PD-1 production [24].

IL-6 also plays an essential role in lung repair responses after viral injury, suggesting that the timing of administration of anti-IL6R could interfere in correct tissue remodeling. In human epithelial cells, SARS-CoV-2 causes a greater production of IL-6 compared to Influenza-A virus and human parainfluenza virus type 2, but with a significantly lower production of SOC3, indicating a possible basis for disproportionate IL-6 responses to this family of viruses [25].

### 2.3. Cytokine Storm

Most critically ill and deceased patients did not show serious clinical symptoms in the early stages of the infection. Most patients only displayed cough, mild fever, or muscle ache. Clinical conditions of these subjects worsened unexpectedly in the later stages of the disease. Acute respiratory distress syndrome (ARDS) and multiple-organ failure occurred precipitously, resulting in death in a short time. It has been speculated that when the body is unable to perform an adequate adaptive immune response against the infection, an innate relentless inflammation can then cause a cytokine storm with ARDS and organ failure [26].

Hence, cytokine storm has a critical role in the process of disease worsening. Therefore, controlling the cytokine storm is an essential method of avoiding the worsening of infected subjects and saving their lives [27,28,29].

Apart from the increase in previously described cytokines, studies of particular interest are related to the cytokines implicated in Th17 responses. IL -1β and TNF-α both increase Th17 responses and vascular permeability and leakage. Th17 cells themselves generate IL-17, IL-21, IL-22 and GM-CSF. IL-17 exerts pro-inflammatory actions by promoting the production of inflammatory cytokines such as IL-1β, IL-6, G-CSF, TNF-α, and chemokines such as IL-8, IP10, KC, MIP2A, MIP3A capable of recruiting more immune cells, and matrix metalloproteinases that contribute to tissue injury and remodeling. IL-17 and TNF-α are able to increase the production of mucins, serum amyloid A, fibrinogen, and anti-apoptotic proteins [30]. The reason for the very high expression of IL-22 in infected 16HBE cells remains unclear. This interleukin has essential functions in tissue repair and immune regulation. Determining whether this high level of expression might explain why virus-infected cells maintain their structure and morphology or whether it might be part of the viral strategy to maintain its transmission ability needs further study [31,32]. 

**Figure 1:**
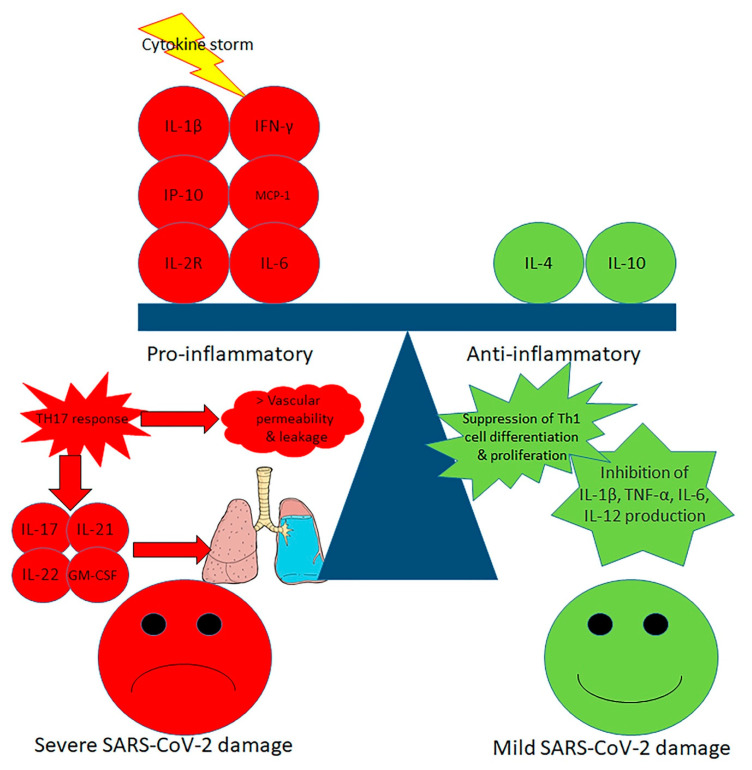
Mechanisms of cytokine storm in severe SARS-CoV-2 infection. In severe Severe Acute Respiratory Syndrome Coronavirus 2 (SARS-CoV-2), a pro-inflammatory cascade is prevalent, with T-helper type 1 (Th1) and T-helper type 17 (Th17) cytokine upregulation leading to increased vascular permeability and leakage with severe lung damage. In mild SARS-CoV-2, an anti-inflammatory behavior is prevalent, featuring the suppression of Th1 cell differentiation and proliferation with inhibition of IL-1β, TNF-α, IL-6 and IL-12 production.

Xu et al. reported that peripheral blood of a subject with grave SARS-CoV-2 infection had an extremely high amount of CCR6 Th17 cells, further sustaining a Th17-type cytokine storm in this infection [33]. Increased Th17 responses were also described in MERS-CoV and SARS-CoV-infected subjects, and in these diseases greater IL-17 concentration with lower IFNγ and IFNα favors worse prognosis [34]. H1N1 influenza virus also caused important Th17 and Th1 responses [35]. In an in vivo experimental model, H1N1 induced an acute IL-17-dependent lung injury [36,37]. Acting on the Th17 pathway may be a successful therapeutic strategy in the SARS-CoV-2-infected subjects with Th17-dominant immune profiles (Figure 1).

Cytokine storm and in particular the huge local production of cytokines is the key element that determines the intensity of symptoms, the mortality rate and the onset of extrapulmonary involvement during SARS-CoV-2 infection [38,39,40]. Critically ill SARS-CoV-2-infected patients demonstrate features suggestive of a group of pathologies collected under the name of cytokine storm syndromes, in which multi-organ failure and hyperinflammation result from an exaggerated cytokine release from an uninhibited immune activation [41]. Comparative studies with these syndromes are stimulating and can offer useful pathogenetic information and effective therapeutic indications.

Actually, the cytokine storm of COVID-19 patients closely resembles the cytokine release syndrome (CRS), a widespread inflammatory condition, which can be induced by drugs and by infection [29,42,43] and is particularly frequent in the course of autoimmune diseases (i.e., juvenile idiopathic arthritis, adult-onset Still’s disease and systemic lupus erythematosus) and immune-related treatment, such as CAR-T cell therapy and organ transplantation [28,44]. Due to the action of pro-inflammatory proteins, vascular permeability augments and a great amount of fluid enters the alveoli, causing dyspnea and respiratory failure [45,46]. The therapeutic approaches used in the treatment of the various CRSs could be usefully evaluated for the cytokine storm of SARS-CoV-2 infection.

## 3. Immunological Alterations and Prognostic Factors

The possibility of estimating the evolutionary trend and the final outcome of the infection at an early stage of the disease and consequently starting an early and effective therapy for those who may progress into a more critical condition could reduce the mortality rate. Ascertaining trustworthy indicators of disease gravity and, above all, reliable markers of a possible negative progression is therefore essential.

It was demonstrated that the CD4+ T cell and CD8+ T cell amounts were strictly correlated to infection gravity and prognosis: the lower the amounts of T cell, CD4+ T cell, and CD8+ T cell at the time of hospitalization, the more negative the prognosis [11,13]. 

However, these findings were only partially confirmed by other groups reporting no significant variation of CD4+ T at the time of admission compared to before discharge, while CD8+ T cells significantly grew in patients with mild severity during hospitalization [47,48].

Other studies have tried to recognize suitable prognostic markers by assessing cytokine levels. High levels of IL-6 were found in severe subjects. Critical SARS-CoV-2 patients had significantly greater concentrations of Th1 cytokines (IL-6 and TNF-α) and greater percentages of ARDS, compared to less severe patients [49]. Significantly higher increases were reported for IL-6 and serum ferritin in non-survivors compared to survivors. These increases, along with the augment of CRP, could indicate the onset of a general inflammatory syndrome capable of causing an acute pulmonary damage that progresses to multiple organ failure [50]. 

Infected subjects with critical disease also have elevated IL-10 serum concentration. This could be a possible compensatory anti-inflammatory effect, in turn responsible for a greater number of secondary infections described in non-survivors [51].

Other parameters such as age, high body mass index, and the increase in transaminases, LDH, soluble IL-2 receptor, and D-Dimer [52,53,54,55] have been identified to be related to ICU admission or death. Severe SARS-CoV-2 infection is frequently complicated with coagulopathy and thrombo-embolic events [56]. Dehydration, multiple cardiovascular risk factors such as diabetes, obesity or hypertension, previous coronary artery disease, ischemic stroke, peripheral artery disease, and classical genetic thrombophilia, such as heterozygous Factor II and Factor V Leiden mutation, are frequent comorbidities in SARS-CoV-2-hospitalized subjects, which, along with the acute inflammatory condition and the protracted immobilization, possibly increase embolic risk. Some authors reported that severely ill COVID-19 patients with multisystem thrombosis and ischemic strokes were found to have antiphospholipid antibodies (aPLAb). However, aPLAb can transiently arise during acute infection, inflammation, or thrombosis. This is the reason for the 12-week interval recommended by ISTH guidelines for confirmatory laboratory testing to diagnose the antiphospholipid syndrome. Actually, there are only very limited data on aPLAb in COVID-19 and it is unclear if they are actually involved in any coagulopathies observed in COVID-19 or represent an epiphenomenon.

In a retrospective analysis, almost half of the patients with laboratory-confirmed SARS-CoV-2 infection had an increase in D-dimer, and the increase was more prominent in more serious patients. The authors suggest that D-dimer alteration can indicate the severity of the infection and an increased concentration is correlated with a poorer prognosis [57]. A different retrospective analysis performed in China demonstrated that prothrombin time (PT) and D-dimer concentrations were greater at the time of admittance of infected patients necessitating ICU assistance, whereas increased D-dimer concentrations were also connected with death in the multivariable analysis [58]. 

In the analysis performed by Tang et al., patients who died had substantively greater fibrin degradation products (FDP) concentrations, augmented PT and activated partial thromboplastin time (aPTT) at the time of admittance to hospital compared to survivors, with a reduction in fibrinogen and antithrombin (AT III) levels [59]. In a prospective study, D-dimer and FDP concentrations were significantly greater in patients with SARS-CoV-2 than in uninfected subjects, and patients with more serious disease had higher values than patients with mild symptoms [60]. 

In an ample meta-analysis, Henry et al. evaluated the prognostic value of hematologic or immunologic markers in subjects with different degrees of disease severity. Infected subjects with critical or fatal disease had elevated white blood cell (WBC) count, and low levels of lymphocytes and platelets compared to less serious infection and survivors. Markers of inflammation, and of cardiac, liver or kidney damage were also significantly higher in subjects with critical or fatal COVID-19 compared to mild disease [61]. 

Numerous reports have confirmed the number and type of leukocytes as appropriate prognostic factors. Indeed, the total WBC count positively correlated with the severity of the disease and the highest values were observed in subjects who died. The increase in WBCs was mainly caused by neutrophils, while a decreasing trend has been observed for lymphocytes, eosinophils and monocytes. Studies on SARS-CoV infection showed that lymphocytes are essential for virus clearance [62], therefore, the observed lymphopenia during COVID-19 infection is a negative prognostic factor, the survival of these patients being dependent on the capability to replace lymphocytes, which are destroyed by the virus [63]. As such, lymphocyte evaluation, specially CD4, may be useful as predictor of gravity and outcome, in fact, patients with more severe clinical illness, or patients who died, had significantly more profound CD4+ lymphopenia.

Mo et al. evaluated 155 SARS-CoV-2 subjects and confirmed that refractory subjects had a greater neutrophil count compared to the most severe patients [64]. Interestingly, some authors studied the clinical features of the SARS-CoV-2 reactivation showing that lymphopenia and neutrophilia are associated with this condition [65].

Neutrophil-to-Lymphocyte ratio (NLR) and Lymphocyte-to-C-reactive protein ratio (LCR) are also recognized inflammation indicators [66,67]. High NLR is a risk marker of mortality not only in infections but also in cancer, coronary syndrome, polymyositis and dermatomyositis [68,69,70,71]. 

The increased NLR is due to an increase in neutrophil count and a reduction in lymphocytes. Inflammation could be responsible for the increased production of neutrophils and might increase the programmed cell death of lymphocytes [72]. 

Multivariate analysis revealed that there was 8% greater risk in mortality for each unit increase in NLR. The NLR of subjects in the highest tertile had a 15.04-fold greater risk of death compared with subjects in the lowest tertile [73]. These data were confirmed by other authors [10,74].

A recent meta-analysis demonstrated that LCR might also be a useful marker of clinical severity in SARS-CoV-2 infection as LCR values were significantly decreased in serious SARS-CoV-2 patients [74].

A further prognostic factor for COVID-19 severity has been identified in the number of circulating platelets, which have an essential role in innate immunity and inflammatory response [75].

Platelets are generated by megakaryocytes in the bone marrow, and recent reports have demonstrated that several cytokines, comprising IL-3, IL-6, IL-9, IL-11, thrombopoietin (TPO), and Stem Cell Factor (SCF), can stimulate the generation of megakaryocytes. In an inflammatory condition, IL-6 can stimulate the production of megakaryocytes by increasing the production of TPO [76,77].

Platelets are present in an inactive form that can be quickly activated at the site of vascular damage, in response to proinflammatory cytokines or infection. Platelets activation is able to induce the release of other cytokines and chemokines leading to a perpetuation of inflammation [78].

Therefore, platelet count can be employed as a sensitive marker of infection and inflammation [79,80] and the platelet-to-lymphocyte ratio (PLR) indicates the intensity of systemic inflammation. Previous reports have shown that PLR is strictly correlated to cancer outcome and severity of diabetes and connective tissue diseases, and can be utilized as a potential inflammatory marker in patients with community-acquired pneumonia [81].

There are relevant connections between platelets and lymphocytes. In fact, platelet-released platelet factor-4 can inhibit agglutinin-A from inhibiting lymphocyte generation, and activated platelets increase lymphocyte adhesion to the endothelium, thus stimulating lymphocyte homing in endothelial veins and their passage to inflammatory sites. PLR mirrors both aggregation and inflammation and may be more suitable in predicting disease outcome than platelet or lymphocyte counts alone [82].

In any case, the use of biological parameters as prognostic factors for COVID-19 severity is a very demanding challenge. In a recent meta-analysis by Wynants et al., 4909 titles were screened, and 51 studies describing 66 prediction models were included [83]. Three different models were recognized for predicting hospital admission from SARS-CoV-2 pneumonia; 47 diagnostic models for identifying SARS-CoV-2 infection; and 16 prognostic models for predicting death risk, evolution to critical disease, or duration of hospital stay. The most stated markers of SARS-CoV-2 infection included fever, age, and symptoms. The most stated markers of bad outcome comprised sex, age, data from computed tomography scans, CRP, LDH and lymphocyte count. 

However, all articles included in the meta-analysis were at high risk of bias, generally because of non-representative selection of control subjects, and at high risk of model overfitting. Moreover, the majority of studies did not contain a description of the study population and calibration of predictions was infrequently considered [83].

Prediction models for SARS-CoV-2 infection are rapidly made available to assist medical decisions. However, the paper of Wynants indicates that proposed models have to be confirmed and new studies are needed.

## 4. Immunological Approach to the Treatment of SARS-COv-2 Infection

**Figure 2:**
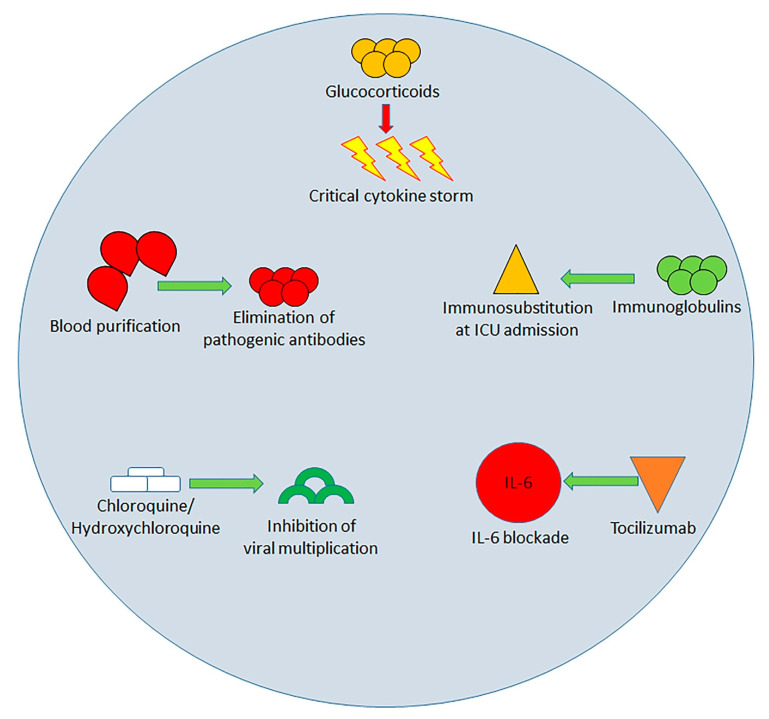
Possible treatment methods for SARS-CoV-2 until present (red arrows indicate inhibition, green arrows indicate promotion/enhancement of the related mechanism). Glucocorticoids limit the production and damaging effects of cytokines so avoiding acute lung injury induced by the cytokine storm; the blood purification removes pathogenic antibodies; Intravenous administration of immunoglobulins (IVIG) has the dual actions of immune substitution and immunomodulation, which are particularly useful during early phase of the cytokine storm; Chloroquine and hydroxychloroquine inhibit viral multiplication and restore the CD8+ cytotoxic viral response; Tocilizumab blocks IL-6; the preliminary results of clinical trials all demonstrate its clinical efficacy.

The identified immunological changes triggered by SARS-COv2 infection represent specific targets of therapies aimed at limiting the spread of the infection and the progress of organ damage. Numerous drugs or drug associations have been used or proposed (Figure 2).

The use of glucocorticoids in treating COVID-19 has become a challenge for clinicians [84]. The scheduling of administration and the dose are critical in severe patients. A premature and/or abundant administration of steroids can inhibit T cells and block B cell antibody production, thus favoring an increased plasma viral load and causing relevant side effects. Therefore, glucocorticoids should be essentially employed in critical subjects experiencing a cytokine storm to limit the production and damaging effects of cytokines, thus avoiding the occurrence of ARDS. Administration of glucocorticoid for 3–5 days, at the dose equivalent to methylprednisolone 1–2mg/kg/day, is indicated for subjects with progressive respiratory failure and rapid imaging progression [85]. It has been shown that ARDS patients treated with methylprednisolone showed reduced mortality compared to those who did not receive glucocorticoids (46% vs. 62%) [86]. 

A different or concomitant therapeutic approach is the intravenous administration of immunoglobulins (IVIG)**.** It was reported that 27% of 99 Wuhan patients received IVIG therapy. This treatment has the dual actions of immune substitution and immunomodulation. IVIG therapy performed within 48 h of entrance to the ICU has been reported to decrease the need for mechanical ventilation and mortality of patients with SARS-CoV-2 pneumonia [87]. However, its value in the therapy of SARS-CoV-2 needs further validation. 

A promising therapeutic approach for patients with severe SARS-CoV-2 infection can be the use of drugs blocking IL-6. Tocilizumab definitely has this action [88]. Clinical evaluations performed in China showed that Tocilizumab is efficacious in critically ill subjects with diffuse bilateral lung alterations, who have increased IL-6 concentrations. The observation was confirmed by an Italian study reporting good results with the use of tocilizumab in patients with SARS-CoV-2 infection [89].

Chloroquine and its less toxic derivative hydroxychloroquine are additional drugs employed in the treatment of COVID-19. Their administration was followed by a reduction in viral proliferation [90]. These drugs act inhibiting viral multiplication in the endosome by avoiding endosomal acidification and endolysosomal fusion. Moreover, hydroxychloroquine stops the entrance of the virus into cells. Furthermore, chloroquine and hydroxychloroquine can inhibit TLR7 and TLR9 signaling and, thus, can restore the CD8+ cytotoxic viral response [91,92]. Adding azithromycin, able to block IL-6 and TNF-alpha [93], further reduces the nasopharyngeal SARS-CoV-2 presence.

Chloroquine phosphate has only been employed in adult patients [94,95]. However, Mehra et al. were unable to confirm a benefit of hydroxychloroquine or chloroquine, when used alone or with macrolide, on in-hospital outcomes for COVID-19 [96].

A completely different type of treatment of the cytokine storm is constituted by blood purification therapy. The capability of blood purification treatment in blocking IL-6/IL-6-receptor-eliminating pathogenic antibodies or cytokines has been demonstrated in this condition [97]. The blood purification therapy, comprising plasma exchange, perfusion, adsorption, and filtration, can be effective in eliminating inflammatory agents to a certain extent. This technique can stop the cytokine storm, thus decreasing the organ damage. It might be employed in critical subjects in the early and middle phases of the infection. The artificial liver treatment can remove inflammatory elements and was employed to block the cytokine storm of H7N9. Its use on SARS-CoV-2 has also attained evident effectiveness [98]. Early renal replacement therapy, which is analogous to the treatment with artificial liver technique, appears to be a useful method to treat cytokine storms [99].

## 5. Potential Targets for Therapeutic Intervention in SARS-COv2 Infection 

Other treatments have been proposed but not yet utilized in SARS-COv-2 patients. 

IFN-λ has antiviral function as it stimulates the expression of antiviral genes in epithelial cells and decreases the mononuclear macrophage-mediated proinflammatory action of IFN-αβ [100], without the inflammatory actions of Type I IFNs. In fact, IFN-λ blocks the passage of neutrophils to the sites of inflammation [101]. Early treatment with IFN had specific effects in decreasing viral load and reduced the clinical symptoms in the Middle East respiratory syndrome coronavirus infection, while, it failed in decreasing the mortality rate [102,103,104]. On the contrary, the late use of interferons did not cause more advantages than placebo [105].

Experiments in in vivo animal models have demonstrated that TNFs contribute considerably to lung damage and alter the T cell response in SARS-CoV-challenged mice. In animals, blocking TNF action or loss of TNF receptor reduced SARS-CoV-caused mortality [106]. However, TNF has not been identified in the serum of SARS-CoV-2 patients in the later phases of the disease and, at present, TNF blockers have not been proposed for subjects with SARS-CoV-2 infection.

Ulinastatin, a natural anti-inflammatory molecule, could have great prospects in the therapy of SARS-CoV-2 infection, as it protects the vascular endothelium by blocking the generation and the release of inflammatory substances. It decreases the concentration of TNF-α, IL-6, and IFN-γ, and augments the concentrations of the anti-inflammatory cytokine IL-10 [107]. Ulinastatin is commonly used to treat pancreatitis and acute circulatory failure.

Sphingosine-1-phosphate (S1P) is a bioactive signaling lysophospholipid that stimulates cytokine production and release [108]. The S1P receptor signaling has the ability to significantly limit immunopathologic injury caused by the host’s innate and adaptive immune response, thereby significantly reducing morbidity and mortality associated with influenza virus infection [109]. Agents able to block Sphingosine-1-phosphate receptor 1 (S1P1) reduce the disproportionate enrolment of inflammatory cells, decrease the amount of proinflammatory cytokines, and diminish the mortality of influenza virus infection [110]. Therefore, S1P1 agonist therapy, such as Siponimod, may be a possible therapeutic choice to block the cytokine storm and clinical trials should be performed to definitely evaluate its therapeutic effectiveness.

Carbajo-Lozoya et al. have reported that an active immunophilin pathway positively influences intracellular Coronavirus replication and that Tacrolimus, an agent that can inhibit calcineurin only when it binds with the immunophilin, intensely blocks the replication of SARS-CoV, HCoV-229E, and HCoV-NL63 at low concentrations in cell cultures [111]. Based on these results, investigating the therapeutic activity of low doses of tacrolimus towards SARS-CoV-2 can also be useful.

The immunomodulatory action of Mesenchymal stem cells (MSC) has been exploited recently for controlling diseases associated with inflammation. In fact, MSC can reduce the altered activation/maturation of T lymphocytes (in particular T-17) and macrophages and favor their differentiation into Treg cells and anti-inflammatory macrophages. They can also inhibit the production of pro-inflammatory cytokines, such as, IL-1, TNF-α, IL-6, IL-12, and IFN-γ, and therefore could be effective in conditions such as the cytokine storm [112,113]. Moreover, their ability to promote IL-10 production and to secrete multiple paracrine factors that regulate endothelial and epithelial permeability, inflammation and improve tissue repair, makes MSCs a possible therapeutic tool for ARDS [114].

A further therapeutic opportunity could be represented by the Inhibitors of mononuclear macrophage recruitment and function. Autopsy findings from patients who died of SARS-CoV2 showed a large amount of mononuclear inflammatory cells infiltrating the lung [33]. Therefore, a possible therapeutic attempt could be to reduce the enrolment of macrophages to the places of inflammation through small interfering RNA-mediated silencing of C-C chemokine receptor type 2, which has been proven to improve the prognosis of inflammatory diseases in animal experiments [115,116]. 

As reported in the previous sections, hyperinflammation is a characteristic of the most severe SARS-CoV 2 infections, therefore treating these patients with cytokine inhibitors to reduce the exaggerated immune response could give positive results [117]. Together with the use of IL-6 inhibitor, now in clinical practice, other anti-cytokine approaches have been evaluated including IL-1 and IL-18 antagonists.

The rationale for the use of these molecules during the cytokine storm of SARS-CoV-2 infection derives from the analogies with other conditions characterized by similar immune reactions. These include the hemophagocytic lymphohistiocytosis (HLH), a rare immune activation syndrome [118,119] and, in particular, the macrophage activation syndrome (MAS), a type of HLH, caused by viral infections in one third of affected patients [120]. MAS is a cytokine storm characterized by a huge release of proinflammatory mediators, with widespread hyperinflammation and progressive multiorgan failure. The suggested molecular mechanism hypothesizes alterations in lymphocytic cytolytic action triggering a pro-inflammatory cytokine cascade [121,122] with high concentrations of IL-1, IL-6, IL-18, soluble IL-2 receptor, TNF, and INF-γ [123] and activation of macrophages leading to tissue hemophagocytosis [124,125].

Anakinra, a recombinant interleukin-1 (IL-1) receptor antagonist, is presently FDA-accepted for the therapy of rheumatoid arthritis and neonatal-onset multi-organ inflammatory disease and off-label used for treating a variety of autoinflammatory diseases, including MAS, with favorable results. In a recent study, continuous i.v. anakinra infusions induced a rapid clinical response in 4/5 severely ill MAS patients with cytokine storm who were refractory to all other treatments [126].

The observation that patients with critical SARS-CoV-2 have shown signs of hemophagocytosis in lung tissues [127], and that Anakinra is effective in the treatment of cytokine storm caused by critical sepsis [128], justifies conducting trials investigating the use of Anakinra in patients with SARS-CoV-2 infection. At present, 15 trials have already been registered at www.ClinicalTrials.gov on the use of Anakinra alone or in combination with other medicines (e.g., ruxolitinib or baricitinib) in COVID-19 patients.

A further therapeutic option for treating the cytokine storm in SARS-CoV-2 patients is represented by the JAK-STAT inhibitors. It is well known that JAK1 and JAK3 pathways are involved in modulating the function of numerous cytokines that are implicated in antiviral responses and in the cytokine storm. Remarkably, IL-6 and GM- CSF, which are both stimulated by SARS- CoV-2, fully depend on JAK2 signaling. STAT3, a transcription factor, mediates IL-6 and IL-23 signals, while both IL-6 and IL-23 stimulate STAT3 via JAK2, and IL-21 stimulates STAT3 via JAK1 and JAK3. 

It has been hypothesized that a JAK2 block can inhibit viral entry of SARS- CoV-2 and the subsequent inflammation [129]. The possible options are using the JAK inhibitors Fedratinib [130] and Baricitinib [129] that are associated with IRE1α Inositol-requiring transmembrane kinase/endoribonuclease 1α (IRE1α), or tylophorine-based compounds [131]. Baricitinib is a JAK inhibitor that has emerged as effective in the therapy of severe rheumatoid arthritis. IRE1α, an endoplasmic reticulum stress sensor, causes an augmented production of the negative controllers of JAK-STAT suppressor of cytokine signaling (SOCS)-1 and SOCS-3 [132]. Tylophorine-based compounds have powerful anti-coronaviral activities against SARS-CoV and MERS-CoV [133] and synergize with JAK2 inhibitors in inhibiting NF-κB activation [134]. 

## 6. Conclusions

Despite the numerous studies produced in a very short time on the pathophysiology and therapy of SARS-COv-2 infection, there are still many shadow areas. Particular attention should be paid to factors that promote/facilitate infection, for example, the age of the infected subjects. Indeed, immune senescence appears to have an essential role in disease progression [135,136].

Aging of the human immune system is characterized by an increase in innate immune responses and chronic inflammation (inflammaging), and a decrease in adaptive responses (immunosenescence). The inflammaging can lead to tissue damage and therefore reduce their ability to resist infections. Immunosenescence limits the ability to respond to new infections like this new coronavirus. This also limits the ability to respond to the vaccine with age.

Treatment is another area of uncertainty: when to perform it, with which drugs, and for how long? The possible risks and benefits of cytokine blockade require a cautious assessment to decide whether and when to start, continue or stop such treatments. Although the reduction in cytokines could be considered “immune suppression” and therefore dangerous in the context of an infection, several molecules, controlling the inflammation induced by the cytokine storm, could be decisive for the survival of the patients [137].

Knowing how to control and modulate the immune response can be a vital factor in controlling disease progression in SARS-CoV-2 infection.
